# Evaluation of the clinical application of histamine H2 receptor antagonists in the prevention of chemotherapy-induced nausea and vomiting based on the attribute hierarchy model method

**DOI:** 10.3389/fphar.2025.1700075

**Published:** 2025-11-25

**Authors:** Jiawei Song, Li Liu, Bingbing Meng, Huanhuan Zhao, Pengcheng Zhang, Huijuan Chen, Yuanyuan Wang

**Affiliations:** 1 Department of Clinical Pharmacy Center, People’s Hospital of Bozhou, Bozhou, China; 2 Department of Medicine, Bozhou Vocational and Technical College, Bozhou, China

**Keywords:** histamine H2 antagonists, chemotherapy-induced nausea and vomiting, attribute hierarchy model, drug utilization evaluation, vomiting

## Abstract

**Objective:**

To investigate the clinical application of histamine H2 receptor antagonists (H2 RAs) in the prevention of chemotherapy-induced nausea and vomiting (CINV), focusing on the rationality of H2 RAs use, the utilization rates and costs of different H2 RAs agents, to provide a reference for the rational use of H2 RAs in this context.

**Methods:**

Evaluation criteria for the rational use of H2 RAs in CINV prevention were established based on domestic and international drug package inserts, clinical guidelines, and national expert consensus. A retrospective study was conducted, and the rationality of H2 RA use in CINV prevention was analyzed and evaluated using the Attribute Hierarchy Model (AHM) method among 323 patients discharged from the People’s Hospital of Bozhou in April 2024. The clinical application of different H2 RAs was analyzed.

**Results:**

The significant issues of irrational use of H2 RAs in CINV prevention included inappropriate solvent and incompatibility (71.83%), use without indications (70.59%), inappropriate dosage and administration (45.82%), prolonged duration of therapy (30.65%), lack of laboratory monitoring (24.46%), and unreasonable efficacy evaluation (23.84%). The mean case score was 71.03 ± 11.06, with oncology cases scoring 70.16 ± 11.03 and non-oncology cases scoring 74.81 ± 10.44, indicating a statistically significant difference (*P* < 0.01). The mean cost of H2 RAs in CINV prevention was 114.46 ± 132.64 yuan, accounting for 2.72% of the mean total drug expenditure. Specifically, the average price of H2 RAs was 123.09 ± 139.45 yuan in oncology cases and 70.89 ± 84.64 yuan in non-oncology cases, also with a statistically significant difference (*P* < 0.01). The clinical application of H2 RAs showed a preference for the higher-priced ranitidine (88.54%).

**Conclusion:**

H2 RAs remain widely used in the prevention of CINV; however, the majority of cases (98.45%) demonstrated irrational use, particularly prophylactic administration without appropriate indications. The rationality of H2 RAs use in oncology departments was lower than that in non-oncology departments, while the associated drug costs were higher. Strengthened management of H2 RAs application in CINV prevention is needed to improve the rationality of drug use and to ensure safety, efficacy, and cost-effectiveness for patients.

## 1 Introduction

Chemotherapy-induced nausea and vomiting (CINV) is one of the most common adverse reactions during chemotherapy. Without prophylactic medication, approximately 70%–80% of patients experience nausea and vomiting, which may lead to dehydration, metabolic disorders, impaired self-care ability, nutritional deficiencies, and other adverse outcomes (Chinese Anti-Cancer Association Committee of Clinical Chemotherapy and Chinese Anti-Cancer Association Committee of Supportive Care, 2022; Zhang, 2022), and may even compromise the proper administration of antineoplastic therapy (Chinese Anti-Cancer Association Committee of Cancer Rehabilitation and Palliative Care et al., 2024). Histamine H2 receptor antagonists (H2 RAs) are typically used in the treatment of acid-related gastrointestinal disorders, such as peptic ulcer disease, prophylaxis of stress-induced ulcers, and gastroesophageal reflux disease (Fisher and Le Couteur, 2001; Black et al., 1972). In addition, both domestic and international guidelines have recommended the use of H2 RAs in the prevention of CINV. The 2014 Guidelines for the Prevention and Treatment of Chemotherapy-Induced Vomiting (Yu et al., 2014) recommended H2 RAs for patients undergoing chemotherapy who also presented with gastric comorbidities. The 2018 Shanghai Expert Consensus on the Comprehensive Management of Chemotherapy-Induced Nausea and Vomiting (Shanghai Anti-Cancer Association Committee of Cancer Rehabilitation and Palliative Care, 2018) suggested that H2 RAs could be considered as part of CINV prophylaxis, especially for patients with gastric diseases. The 2022 Chinese Pharmaceutical Association Group Standard: Guidelines for the Prevention and Treatment of Chemotherapy-Induced Nausea and Vomiting (Zhang, 2022) did not recommend routine prophylactic use of H2 RAs before chemotherapy but suggested their use during chemotherapy for the management of heartburn or nausea symptoms. The 2023 Chinese Guidelines for the Prevention and Treatment of Chemotherapy-Related Nausea and Vomiting (Chinese Anti-Cancer Association Committee of Cancer Rehabilitation and Palliative Care et al., 2024) proposed discretionary use of H2 RAs, noting that patients with reflux symptoms may benefit from their inclusion. Similarly, the NCCN Antiemesis Guidelines 2024 Version 1 (National Comprehensive Cancer Network, 2023) suggested that H2 RAs may be considered for patients with reflux symptoms. Over time, guideline recommendations for the use of H2 RAs in CINV prevention have evolved from “recommended” to “discretionary.” Moreover, recent guidelines have further restricted the conditions for their use, shifting from a broad indication of “gastric comorbidities” to a narrower focus on patients experiencing “acid reflux” or “heartburn” symptoms.

The Attribute Hierarchy Model (AHM) is a simple, practical, and effective method for multi-attribute decision-making, supported by scientific theory (Wang and Hou, 2006). In 2021, the present author first applied the AHM method to drug utilization evaluation (DUE), and it has been widely used in recent years for the comprehensive review of the rationality of clinical drug application (Meng et al., 2022; Yang, 2024; Cheng et al., 2024). A literature search of the China National Knowledge Infrastructure (CNKI), Wanfang Med Online, and the Chinese Medical Journal Full-text Database identified only two relevant studies, both published in 2004, concerning the application of H2 RAs in CINV (Zheng et al., 2004a; Zheng et al., 2004b). In this study, DUE criteria for the application of H2 RAs in CINV prevention were developed with reference to clinical guidelines and domestic and international drug package inserts. Based on the AHM method, the rationality of clinical application was comprehensively evaluated, and information such as the average cost per patient for H2 RAs in CINV prevention was obtained, promoting rational drug use and safeguarding patient interests.

## 2 Materials and methods

### 2.1 Data sources

Data were extracted from the Pharm Assist Rational Drug Use System (Sichuan Meikang) for all chemotherapy-related discharge cases from People’s Hospital of Bozhou, a tertiary grade-A general hospital (Anhui Province, China), in April 2024. The inclusion criteria were as follows: cases with diagnostic keywords including “chemotherapy” or “chemical therapy”; patients who received H2 RAs during hospitalization; and cases with complete information available (including medication administration time, dosage and route of administration, admission and discharge diagnoses, medical records, and cost data). Exclusion criteria included: cases enrolled in clinical trials; patients who did not undergo chemotherapy during hospitalization; cases in which radiotherapy was administered during hospitalization; cases in which H2 RAs were not used for the prevention of CINV; cases of voluntary discharge, hospital transfer or death. The inclusion criteria were formulated with reference to the Guidelines for the Prevention and Treatment of Chemotherapy-Induced Nausea and Vomiting (Zhang, 2022) and the Chinese Guidelines for the Prevention and Treatment of Antitumor Therapy–Related Nausea and Vomiting (2023 Edition) (Chinese Anti-Cancer Association Committee of Cancer Rehabilitation and Palliative Care et al., 2024).

This retrospective study aimed to analyze the rational use of H2 RAs.To ensure adequate precision in estimating the proportion of rational drug use, the sample size was calculated using the formula for estimating a single proportion. The calculation formula is as follows:

$$n=\frac{Z_{1–a/2}^{2}\times p\times \left(1–p\right)}{d^{2}}\;$$

where *Z*_1−α/2_ = 1.96 corresponds to a 95% confidence interval, *p* represents the expected proportion of rational drug use, and *d* denotes the desired half-width of the confidence interval. Based on previous literature and pilot observations, the expected proportion of rational use was set at 80% (*p* = 0.80), with a precision of ±0.05 (*d* = 0.05). The initial calculated sample size was approximately 246 cases. Considering a 10% allowance for missing or incomplete data, the final required sample size was approximately 273 cases. This sample size ensures that the estimated proportion of rational drug use has a 95% confidence interval with a margin of error within ±0.05.

### 2.2 Establishment of DUE criteria for H2 RAs in the prevention of CINV

With reference to domestic and international prescribing information (U.S. Food and Drug Administration, 2009; U.S. Food and Drug Administration, 2005; U.S. Food and Drug Administration, 2021; U.S. Food and Drug Administration, 2006; U.S. Food and Drug Administration, 2024; U.S. Food and Drug Administration, 2018), the Guidelines for the Prevention and Treatment of Chemotherapy-Induced Nausea and Vomiting (Zhang, 2022), the Chinese Expert Consensus on the Prevention and Treatment of Nausea and Vomiting Related to Antitumor Drug Therapy (2022 Edition) (Chinese Anti-Cancer Association Committee of Clinical Chemotherapy and Chinese Anti-Cancer Association Committee of Supportive Care, 2022), the Chinese Expert Consensus on the Prevention and Treatment of Delayed Nausea and Vomiting (2022 Edition) (Chinese Anti-Cancer Association Committee of Supportive Care, 2023), the Chinese Guidelines for the Prevention and Treatment of Nausea and Vomiting Related to Antitumor Therapy (2023 Edition) (Chinese Anti-Cancer Association Committee of Cancer Rehabilitation and Palliative Care et al., 2024), the Shanghai Expert Consensus on the Comprehensive Management of Chemotherapy-Induced Nausea and Vomiting (2024 Edition) (Shanghai Anti-Cancer Association Committee of Cancer Rehabilitation and Palliative Care et al., 2024), the NCCN Antiemesis Guidelines 2024 Version 1 (National Comprehensive Cancer Network, 2023), and the National Formulary of China (Volume on Chemical Drugs and Biological Products, Pediatric Edition) (Editorial Board of Chinese National Formulary, 2013), preliminary criteria for drug DUE of H2 RAs in the prevention of CINV were established.

To refine and finalize these criteria, both internal and external experts were invited to review and revise them. The external experts included one chief pharmacist specializing in hospital pharmacy from a tertiary grade-A general hospital, one chief physician of oncology, and four associate chief pharmacists specializing in clinical pharmacy. The internal experts comprised one chief physician and one associate chief physician from the Department of Oncology, one associate chief nurse from the Oncology Department, one associate chief physician from the Department of Hematology, and one associate chief pharmacist specializing in clinical pharmacy. The final DUE criteria were established after consensus was reached, with no objections raised during the revision process.

### 2.3 Calculation of weight coefficients for evaluation indicators based on the AHM

Method Pairwise comparisons were performed among 11 evaluation indicators, including appropriateness of indications, dosage and administration, solvent and compatibility and others, to determine their relative importance and establish the weight of each indicator. The importance of each indicator was quantified numerically for comparative purposes. These numerical values lacked specific units; instead, they represented the relative degree of significance between indicators, with larger values denoting higher importance and smaller values indicating lower importance.

Here, μ_ij_ denotes the importance value of indicator i relative to indicator j; μ_ji_ denotes the importance value of indicator j relative to indicator i; and μᵢᵢ represents the comparison of indicator i with itself, which, according to mathematical rules, is defined as μᵢᵢ = 0, with μ_ij_+μ_ji_ = 1. Taking the evaluation indicators indications and dosage and administration as an example, the sum of their relative importance is assigned a value of 1. The relative importance of indications compared with dosage and administration is 0.8, whereas that of dosage and administration compared with indications is 0.2. The relative importance of indications to itself is 0, and similarly, the relative importance of dosage and administration to itself is also 0. Following the same procedure, pairwise comparisons were conducted among the eleven evaluation indicators, including each indicator’s comparison with itself, to construct the complete judgment matrix for this study. The consistency of the constructed judgment matrix was then tested according to Equation 1, and the relative weight vector of the indicators was subsequently derived using Equation 2 (Song et al., 2022).

$$g\left(x\right)=\left\{\begin{array}{c} 1,x>0.5\\ 0,x\leq 0.5 \end{array}\right.$$

$$\text{Qi}=\left\{j:g\left(\mu_{ii}\right)=1,1\leq j\leq n\right\}$$

$$g\left(\mu_{ik}\right)=g\left[\sum_{jen}g\left(\mu_{ik}\right)\right]\geq 0,1\leq k\leq n$$ (1)

$$w_{c}\left(i\right)=\frac{2}{n\left(n‐1\right)}\sum_{j=1}^{m}\mu_{\text{ij}}$$ (2)

### 2.4 Case evaluation and scoring

The hospital stipulates that cases fully meeting the evaluation criteria are assigned 100 points. The score for each evaluation indicator is calculated by multiplying the relative attribute weight vector of that indicator by 100 points. If any indicator in the case is deemed unreasonable, the corresponding points for that indicator are deducted, resulting in the final score for each case. For example, in the evaluation criteria of this study, there are a total of 11 evaluation indicators, which we label as A, B, C, D, E, F, G, H, I, J and K. The Medical Record Score (MRS) is calculated as follows: MRS = 100-(0 or 1) × Score of Indicator A- (0 or 1) × Score of Indicator B-(0 or 1) × Score of Indicator C-(0 or 1) × Score of Indicator D-(0 or 1) × Score of Indicator E−(0 or 1) × Score of Indicator F-(0 or 1) × Score of Indicator G-(0 or 1) × Score of Indicator H-(0 or 1) × Score of Indicator I-(0 or 1) × Score of Indicator J-(0 or 1) × Score of Indicator K. For each indicator, the multiplier was set to 0 if the criterion was met (rational use) and 1 if the criterion was not met (irrational use). A higher MRS indicated closer adherence to the evaluation standards and thus greater rationality of clinical application. In this study, the research team evaluated the rationality of medication use in all included cases based on the established evaluation criteria. Any inappropriate indicators identified within the cases were recorded and statistically analyzed. When the rationality of a case was difficult to determine due to ambiguous descriptions, the evaluators consulted the prescribing physicians to clarify the clinical context. Subsequently, the team conducted analyses and discussions to reach a consensus judgment. The inappropriate indicators in each case were then quantified, and the MRS for each case was calculated using the MRS formula described above.

### 2.5 Statistical analysis

According to the hospital’s regulations, a case that fully met all evaluation criteria, including Patient age, sex, weight, coefficients, rationality rate, Medical Record Score (MRS), average cost per patient, and the proportion of average cost per patient, was summarized and analyzed using Microsoft Excel 2010. Statistical analyses were performed with SPSS Statistics 23.0 (IBM Corp., Armonk, NY, United States). Categorical variables were expressed as cases (percentage), while continuous variables conforming to a normal distribution were presented as mean ± standard deviation (SD). Comparisons between groups were conducted using the independent samples t-test, and a two-sided *P* value of less than 0.05 was considered statistically significant.

## 3 Results

### 3.1 DUE criteria for H2 RAs in the prevention of CINV

The revised and finalized DUE criteria for H2 RAs in the prevention of CINV are presented in Table 1.

**TABLE 1:** Evaluation criteria for drug use of H2 RA in CINV drug prevention.

Indications	H2 RA are not routinely used for the pharmacological prevention of chemotherapy-induced nausea and vomiting (CINV), unless the patient presents with clinical symptoms of gastroesophageal reflux, acid regurgitation, or heartburn prior to the initiation of chemotherapy (Zhang, 2022; Chinese Anti-Cancer Association Committee of Cancer Rehabilitation and Palliative Care et al., 2024)	
	If a patient experiences breakthrough or refractory vomiting despite prophylactic antiemetic therapy for CINV, an H2 RA may be added as part of the pharmacological management of CINV (Chinese Anti-Cancer Association Committee of Cancer Rehabilitation and Palliative Care et al., 2024; Chinese Anti-Cancer Association Committee of Supportive Care, 2023)	
Dosage and administration	Cimetidine	Adults: 0.2 g twice daily orally (po)/0.2–0.4 g once daily intravenously (iv) (Shanghai Anti-Cancer Association Committee of Cancer Rehabilitation and Palliative Care et al., 2024)
		Children (≥12 years): 0.2 g once daily; maximum daily dose 0.4 g (Editorial Board of Chinese National Formulary, 2013)
	Ranitidine	Adults: 0.4 g twice daily orally (po)/50 mg twice daily intravenously (iv) (Shanghai Anti-Cancer Association Committee of Cancer Rehabilitation and Palliative Care et al., 2024)
		Children (≥8 years): total daily dose 2–4 mg/kg, administered intravenously in 3–4 divided doses; maximum single dose 50 mg (U.S. Food and Drug Administration, 2009)
	Famotidine	Adults: 20 mg twice daily orally (po)/20 mg twice daily intravenously (iv) (Shanghai Anti-Cancer Association Committee of Cancer Rehabilitation and Palliative Care et al., 2024)
		Children: 0.4 mg/kg twice daily orally (po) or intravenously (iv)
Solvent and compatibility	Cimetidine	0.2 g reconstituted with 0.9% normal saline (NS), glucose solution (GS), or glucose–sodium chloride injection; for intravenous injection (iv), use 20 mL; for intravenous infusion (ivgtt), use 250–500 mL
	Ranitidine	Prepared with 200 mL of 5% glucose solution (GS)
	Famotidine	For intravenous injection (iv): reconstituted with 20 mL of 0.9% normal saline (NS) or 5% glucose solution (GS); for intravenous infusion (ivgtt): reconstituted with 250 mL of 5% GS
Injection time	Cimetidine	For intravenous injection (iv): 2–3 min; for intravenous infusion (ivgtt): 1–4 mg/kg/h
	Ranitidine	For intravenous injection (iv): >10 min; for intravenous infusion (ivgtt): 1–2 h
	Famotidine	For intravenous injection (iv): >3 min; for intravenous infusion (ivgtt): >30 min
Duration of therapy	The duration of intravenous infusion should not exceed 5 days; switch to oral administration if the patient’s condition permits	
	Discontinue medication promptly once symptoms such as gastroesophageal reflux, acid regurgitation, or heartburn improve	
Concomitant medication	H2 RAs	Not recommended in combination with gefitinib; if concomitant use is necessary, administer H2 RAs 6 h before or 6 h after gefitinib (U.S. Food and Drug Administration, 2005)
		Not recommended in combination with neratinib; if concomitant use is necessary, administer H2 RAs 2 h before or 10 h after neratinib (U.S. Food and Drug Administration, 2021)
		Not recommended in combination with dasatinib; if concomitant use is necessary, short-acting antacids should be used instead of H2 RAs with an interval of several hours (U.S. Food and Drug Administration, 2006)
		Avoid combination with tepotinib; if unavoidable, administer H2 RAs 2 h before or 10 h after tepotinib (U.S. Food and Drug Administration, 2024)
		Avoid concomitant use with other H2 RAs
		Avoid concomitant use with proton pump inhibitors (PPIs) (National Health Commission of the People’s Republic of China, 2021)
	Cimetidine	Avoid concomitant use with central anticholinergic agents
		Avoid concomitant use with carmustine, fluorouracil, or epirubicin
		Contraindicated with caffeine or dofetilide
		When combined with warfarin, monitor the international normalized ratio (INR)
	Ranitidine	Avoid concomitant use with tizanidine
	Famotidine	Not recommended in combination with delavirdine mesylate, cefditoren, or fosamprenavir (U.S. Food and Drug Administration, 2018)
Special populations	Elderly	Dose reduction or extension of dosing interval
	Pregnancy	Contraindicated
	Lactation	Contraindicated
	Children	Cimetidine is not recommended for children <12 years; ranitidine is contraindicated for children <8 years
	Renal impairment	Ranitidine requires dose reduction with plasma drug concentration monitoring; famotidine is contraindicated in patients with severe renal impairment
	Hepatic impairment	Ranitidine requires dose reduction with plasma drug concentration monitoring; famotidine is contraindicated in patients with severe renal impairment
Contraindications	Cimetidine	Acute pancreatitis; hypersensitivity to cimetidine
	Ranitidine	Hypersensitivity to ranitidine, anhydrous citric acid, or disodium hydrogen phosphate dodecahydrate; acute porphyria
	Famotidine	Hypersensitivity to famotidine or other H2 receptor antagonists
Laboratory monitoring	Cimetidine	At least one assessment of liver function, renal function, and complete blood count (CBC) after drug administration
	Ranitidine	At least one assessment of renal function, aspartate aminotransferase (AST), and alanine aminotransferase (ALT) after drug administration
	Famotidine	At least one assessment of complete blood count (CBC) after drug administration
Adverse reactions	No adverse reactions occurred or relevant information was unavailable	
	If adverse reactions occur: evaluate, analyze, manage, and report as required	
Efficacy assessment	Not evaluated	
	Evaluated as effective: continue treatment according to the prescribed course	
	Evaluated as ineffective: switch to another drug or adjust dosage and administration	

### 3.2 Case characteristics

This study estimated the required sample size to be 273 cases. A total of 361 cases were initially enrolled according to the inclusion criteria. After applying the exclusion criteria, 38 cases were excluded, resulting in 323 cases being ultimately evaluated. The actual sample size for evaluation exceeded the estimated sample size. Among them, 139 were female (43.03%) and 184 were male (56.97%). The mean age was 64.58 ± 10.89 years (range: 26–90 years).

The departmental distribution of cases was as follows: 106 cases (32.82%) from Oncology Department Ward I, 63 cases (19.50%) from Oncology Ward II, 94 cases (29.72%) from Oncology Ward III, 19 cases (5.88%) from Respiratory and Critical Care Medicine Ward I, 13 cases (4.02%) from Respiratory and Critical Care Medicine Ward II, 6 cases (1.86%) from Respiratory and Critical Care Medicine Ward III, 7 cases (2.17%) from Hematology Ward I, 5 cases (1.55%) from Hematology Ward II, 7 cases (2.17%) from Gastrointestinal Surgery, 2 cases (0.62%) from Gynecology Ward II, and 1 case (0.31%) from the Interventional Ward. All cases were from China.

The mean cost of H2 RAs used for the prevention of CINV was 114.46 ± 132.64 yuan (range: 1.2–1356.6 yuan), accounting for 2.72% (cost of H2 RA/average total inpatient drug cost per patient) of the mean total drug cost. The maximum proportion reached 30.81%, while the minimum was 0.003%. The mean H2 RAs cost was 123.09 ± 139.45 yuan in oncology cases and 70.89 ± 84.64 yuan in non-oncology instances, with the difference being statistically significant (*P* < 0.01).

### 3.3 Weight coefficients of evaluation indicators and irrational use

Five in-hospital experts (one chief physician, one associate chief physician, one associate chief nurse, and two associate chief pharmacists) were invited to construct a judgment matrix based on the AHM. After a thorough discussion, a consensus was reached, and a unified matrix was established. Using the AHM method, weight coefficients were calculated for each of the 11 evaluation indicators—including indications, dosage and administration, solvent and compatibility, among others. Scores were then assigned to each indicator, and all sampled cases were evaluated according to the established DUE criteria, with specific scores calculated for each case. The final expert-reviewed judgment matrix is presented in Table 2, while the number of irrational cases, weight coefficients, and indicator scores are summarized in Table 3.

**TABLE 2:** Construction of evaluation index matrix, n = 11.

Evaluation indicators	Indications	Dosage and administration	Solvent and compatibility	Injection time	Duration of therapy	Concomitant medication	Special populations	Contraindications	Laboratory monitoring	Adverse reactions	Efficacy assessment
Indications	0	0.8	0.8	0.9	0.7	0.75	0.75	0.6	0.8	0.8	0.9
Dosage and administration	0.2	0	0.6	0.6	0.5	0.6	0.7	0.4	0.5	0.7	0.7
Solvent and compatibility	0.2	0.4	0	0.5	0.5	0.5	0.6	0.4	0.5	0.6	0.6
Injection time	0.1	0.4	0.5	0	0.4	0.4	0.4	0.3	0.4	0.4	0.5
Duration of therapy	0.3	0.5	0.5	0.6	0	0.5	0.6	0.4	0.6	0.7	0.7
Concomitant medication	0.25	0.4	0.5	0.6	0.5	0	0.5	0.4	0.6	0.6	0.6
Special populations	0.25	0.3	0.4	0.6	0.4	0.5	0	0.3	0.5	0.6	0.6
Contraindications	0.4	0.6	0.6	0.7	0.6	0.6	0.7	0	0.7	0.7	0.7
Laboratory monitoring	0.2	0.5	0.5	0.6	0.4	0.4	0.5	0.3	0	0.5	0.5
Adverse reactions	0.2	0.3	0.4	0.6	0.3	0.4	0.4	0.3	0.5	0	0.5
Efficacy assessment	0.1	0.3	0.4	0.5	0.3	0.4	0.4	0.3	0.5	0.5	0

**TABLE 3:** Rationality, weight coefficients, and indicator scores of various evaluation indicators for H2 RA in CINV drug prevention n = 323, Example (%).

Evaluation indicators	Rational (cases)	Irrational (cases)	Weight coefficient	Indicator score
Indications	95 (29.41%)	228 (70.59%)	0.1418	14.18
Dosage and administration	175 (54.18%)	148 (45.82%)	0.1	10
Solvent and compatibility	91 (28.17%)	232 (71.83%)	0.0873	8.73
Injection time	323 (100%)	0 (0%)	0.0691	6.91
Duration of therapy	224 (69.35%)	99 (30.65%)	0.0982	9.82
Concomitant medication	286 (88.54%)	37 (11.46%)	0.09	9
Special populations	314 (97.21%)	9 (2.79%)	0.0809	8.09
Contraindications	323 (100%)	0 (0%)	0.1145	11.45
Laboratory monitoring	244 (75.54%)	79 (24.46%)	0.08	8
Adverse reactions	315 (97.52%)	8 (2.48%)	0.0709	7.09
Efficacy assessment	246 (76.16%)	77 (23.84%)	0.0673	6.73

### 3.4 Medical Record Score

The mean Medical Record Score (MRS) of the included cases was 71.03 ± 11.06 (range: 42.54–100). The mean MRS for oncology departments was 70.16 ± 11.03, whereas that for non-oncology departments was 74.81 ± 10.44, and the difference was statistically significant (*P* < 0.01). Detailed MRS results are shown in Table 4.

**TABLE 4:** Distribution of Medical Record Score (MRS).

Medical Record Score (MRS)	Number of cases (n)	Proportion (%)
MRS = 100	5	1.55
90 ≤ MRS < 100	16	4.95
80 ≤ MRS < 90	38	11.76
60 ≤ MRS < 80	203	62.85
MRS < 60	61	18.89

## 4 Discussion

### 4.1 Major issues of irrational use and proposed solutions

The problem of inappropriate solvent and compatibility was mainly due to the use of 100 mL of solvent, which is lower than the amount recommended in the package inserts, resulting in excessively high drug concentrations and potentially leading to adverse reactions. A clinical case has been reported in which cimetidine prepared with 100 mL of solvent caused severe anaphylactic shock after a 20-min infusion (Peng et al., 2016). The issue of off-label use was reflected in the routine administration of H2 RAs for CINV prophylaxis in patients without symptoms such as acid reflux or heartburn. Recent guidelines have explicitly stated that H2 RAs are not recommended for the routine prevention of CINV (Zhang, 2022; Chinese Anti-Cancer Association Committee of Cancer Rehabilitation and Palliative Care et al., 2024; National Comprehensive Cancer Network, 2023). Inappropriate dosage and administration were manifested as dosing frequencies inconsistent with expert consensus. Frequencies lower than recommended may fail to achieve therapeutic plasma concentrations, prolong treatment duration, and increase drug costs. Conversely, frequencies higher than recommended may elevate the risk of adverse reactions and compromise the safety of drug therapy. In this study, the most extended continuous use of H2 RAs was 57 days. Therefore, the clinical application of H2 RAs should follow package inserts, clinical guidelines, and patient symptoms to ensure safe, effective, and cost-efficient treatment. The lack of laboratory monitoring was mainly observed in patients with baseline abnormalities in liver function, renal function, or blood routine tests upon admission, who did not undergo monitoring during H2 RA use, despite package inserts requiring such monitoring. The problems with efficacy assessment included failure to evaluate the prophylactic effect in patients with reflux symptoms at admission, failure to discontinue H2 RAs promptly after effective symptom relief, and failure to adjust treatment regimens when therapy was ineffective.

Regarding concomitant medication, irrational use was mainly observed in the combination of H2 RAs with proton pump inhibitors for CINV prevention, or in regimens that included epirubicin or fluorouracil combined with cimetidine. For patients with reflux or heartburn symptoms, either an H2 RA or a PPI alone should be used for prophylaxis; except in cases of nocturnal acid breakthrough, the combination of H2 RAs with PPIs is generally not recommended (National Health Commission of the People’s Republic of China, 2021). Moreover, the concomitant use of cimetidine with fluorouracil or epirubicin may enhance chemotherapy-induced bone marrow suppression; in such cases, famotidine or ranitidine may be considered as alternatives.

Secondary irrationalities included the failure to reduce the dose or extend the dosing interval in elderly patients. Adverse reactions were reflected in abnormal liver or renal function values in laboratory tests during treatment, despite being normal at baseline; however, these potential adverse drug reactions (ADRs) were not evaluated, analyzed, or reported as required.

There are considerable problems associated with the use of H2 RAs in the prevention of CINV, and administrative intervention is required to promote rational use. The proposed solutions include the following three aspects. First, strengthen training on CINV prophylaxis by organizing physicians to study relevant diagnostic and treatment standards, as well as clinical guidelines, thereby ensuring strict adherence to the indications for H2RA use in CINV prevention. Second, optimize the pre-prescription review system by refining audit rules in accordance with the hospital’s specific patterns of irrational drug use. For example, in terms of solvent volume selection, orders that do not comply with the solvent volume recommended in the drug package inserts should not be allowed to proceed. Finally, conduct regular evaluations of the rationality of H2 RA use in the prevention of CINV. Based on MRS, various intervention strategies should be adopted, including, but not limited to, financial penalties, public notification and criticism, interviews with prescribers, and restrictions on prescribing rights.

### 4.2 Clinical application characteristics of H2 RAs in the prevention of CINV

The mean MRS in oncology departments was significantly lower than that in non-oncology departments, indicating that the rationality of H2 RA use for CINV prophylaxis was inferior in oncology compared with non-oncology specialties. The clinical application of H2 RAs for CINV prevention exhibited the following characteristics: Inadequate control of indications, with a tendency toward routine prophylactic use. A total of 202 cases (62.54%) received H2 RAs from the first day of admission until discharge, of which 194 cases (96.04%) were from oncology departments. Longer treatment duration in oncology departments. Among non-oncology cases, 93.10% of H2 RA use occurred only during chemotherapy, whereas in oncology departments, only 26.24% of cases received H2 RAs during chemotherapy, and 73.76% were administered H2 RAs continuously from admission until discharge. Preference for ranitidine in drug selection. Of the included cases, 286 involved ranitidine, 24 cimetidine, and 13 famotidine. International data show that in 2019 ranitidine ranked 53rd and famotidine 104th among the top 200 most commonly used drugs (Meng et al., 2023). In this study, 88.54% of cases used ranitidine. The limited use of famotidine may be related to its inclusion in the second batch of the National Key Monitoring Drug List. It is noteworthy that in April 2020 the U.S. Food and Drug Administration (FDA) ordered the market withdrawal of all ranitidine products due to the potential presence of the carcinogen N-nitrosodimethylamine (NDMA).

Neither domestic nor international guidelines (Zhang, 2022; Chinese Anti-Cancer Association Committee of Cancer Rehabilitation and Palliative Care et al., 2024; National Comprehensive Cancer Network, 2023) have provided specific recommendations on the selection of H2 RA agents. In addition to safety and efficacy, cost-effectiveness must also be considered. According to the drug prices in the study hospital, the prophylactic cost per day (drug price × daily dosage) was 47.6 yuan for ranitidine, 3.92 yuan for famotidine, and 0.6–1.2 yuan for cimetidine. Under comparable safety and efficacy, famotidine or cimetidine could be considered as alternatives to reduce economic burden.

### 4.3 Strengths and limitations of this study

This study applied the AHM to comprehensively evaluate the rationality of H2 RA use in preventing CINV. A specific rationality score was obtained for each case, and the distribution of these scores intuitively reflected the overall pattern of drug utilization. By transforming traditional categorical evaluation results into quantifiable scores, the AHM method demonstrated certain advantages. According to the traditional evaluation method, any case presenting at least one inappropriate indicator is classified as an inappropriate case. As shown in Table 4, based on the traditional evaluation method only 5 cases were deemed appropriate (MRS = 100, 1.55%), whereas 318 cases were considered inappropriate (MRS < 100, 98.45%). However, this approach fails to further reflect the degree of inappropriateness. For example, Case A involved inappropriate dosage and administration, whereas Case B exhibited multiple issues, including lack of therapeutic indication, inappropriate dosage and administration, irrational drug combination, and contraindicated medication use. According to the traditional evaluation method, both cases would be classified simply as inappropriate. In this study, however, by applying an attribute hierarchy model to determine the relative weight coefficients of each evaluation indicator, we assigned corresponding scores to each case. The rationality score of Case A was 85.82, while that of Case B was 55.37. These scores provide a clear quantitative representation of the degree of medication rationality, thereby facilitating a more precise understanding of the appropriateness of drug use. In addition, this study provided information on the average cost per patient and its proportion of total drug costs across 323 cases, offering valuable reference data for future research. However, several limitations should be noted. First, the criteria for concomitant drug use with H2 RAs are numerous, especially for cimetidine. The DUE criteria established in this study were based on the oncology department’s formulary, incorporating rules on avoidance and contraindicated combinations, which may limit generalizability. Information on adverse reaction indicators was limited in this study. Only eight cases of adverse reactions were identified, all of which were detected through patients’ laboratory test reports during case evaluation. Laboratory results at the time of hospital admission were within normal ranges, whereas abnormal values were observed after drug administration. Specifically, elevated AST or ALT levels (indicators of hepatic function) were observed in five cases, decreased creatinine levels (an indicator of renal function) in two cases, and decreased uric acid and urea levels in one case. In all eight cases, the physicians failed to perform proper evaluation, analysis, management, and reporting of the adverse reactions as required; therefore, all cases were classified as having irrational management of adverse reactions. However, numerous instances of irrational practices were identified in medication dosage, solvent compatibility, and treatment duration, which may have contributed to an increased incidence of adverse reactions. As this study was retrospective in nature and relied on medical records and laboratory reports, some adverse reactions may not have been documented or were inadequately managed, resulting in incomplete data acquisition. Therefore, the evaluation of the rationality of adverse reaction indicators in this study has certain limitations. Finally, as a single-center study, its findings may not be broadly representative of the general population. Future studies will expand to include multiple centers and apply both univariate and multivariate logistic regression analyses to identify potential factors contributing to irrational drug use, to provide more substantial evidence to improve the rational application of H2 RAs in CINV prevention.

## Data Availability

The original contributions presented in the study are included in the article/supplementary material, further inquiries can be directed to the corresponding author.
